# Molecular Characterization, Protein–Protein Interaction Network, and Evolution of Four Glutathione Peroxidases from *Tetrahymena thermophila*

**DOI:** 10.3390/antiox9100949

**Published:** 2020-10-02

**Authors:** Diana Ferro, Rigers Bakiu, Sandra Pucciarelli, Cristina Miceli, Adriana Vallesi, Paola Irato, Gianfranco Santovito

**Affiliations:** 1 BIO5 Institute, University of Arizona, Tucson, AZ 85719, USA; ferro.d.bio@gmail.com; 2 Department of Pediatrics, Children’s Mercy Hospital and Clinics, Kansas City, MO 64108, USA; 3 Department of Aquaculture and Fisheries, Agricultural University of Tirana, 1000 Tiranë, Albania; rigers.bakiu@ubt.edu.al; 4 School of Biosciences and Veterinary Medicine, University of Camerino, 62032 Camerino, Italy; sandra.pucciarelli@unicam.it (S.P.); cristina.miceli@unicam.it (C.M.); adriana.vallesi@unicam.it (A.V.); 5 Department of Biology, University of Padova, 35131 Padova, Italy; paola.irato@unipd.it

**Keywords:** protein–protein interaction network, GPx, glutathione peroxidases genes, ciliate protists, copper, metals, antioxidant system, free-radicals, ROS, reactive oxygen species

## Abstract

Glutathione peroxidases (GPxs) form a broad family of antioxidant proteins essential for maintaining redox homeostasis in eukaryotic cells. In this study, we used an integrative approach that combines bioinformatics, molecular biology, and biochemistry to investigate the role of GPxs in reactive oxygen species detoxification in the unicellular eukaryotic model organism *Tetrahymena thermophila*. Both phylogenetic and mechanistic empirical model analyses provided indications about the evolutionary relationships among the GPXs of *Tetrahymena* and the orthologous enzymes of phylogenetically related species. In-silico gene characterization and text mining were used to predict the functional relationships between GPxs and other physiologically-relevant processes. The GPx genes contain conserved transcriptional regulatory elements in the promoter region, which suggest that transcription is under tight control of specialized signaling pathways. The bioinformatic findings were next experimentally validated by studying the time course of gene transcription and enzymatic activity after copper (Cu) exposure. Results emphasize the role of GPxs in the detoxification pathways that, by complex regulation of GPx gene expression, enable *Tethraymena* to survive in high Cu concentrations and the associated redox environment.

## 1. Introduction

The maintenance of an equilibrium between production and elimination of reactive oxygen species (ROS) is a fundamental molecular process that has been playing a pivotal role in eukaryotic survival and aging since the appearance of the aerobic metabolism [1]. In nature, ROS are continuously generated in living organisms as a normal consequence of electrons moving across the inner mitochondrial membrane and free oxygen that is present inside the cell. ROS formation can also be increased as a result of exogenous and endogenous stressors, such as exposure to environmental contaminants, treatment of pathologic conditions with drugs, and onset or progression of diseases [2,3,4,5]. In order to modulate ROS presence, antioxidant molecules are present in cells as a defense against the risk of oxidative stress [6]. Antioxidant enzymes and metabolites should continuously and efficiently work together since small variations in their physiological concentrations can have dramatic effects on the resistance of cellular lipids, proteins, and DNA to oxidative damage. As a consequence, the antioxidant system is characterized by strong protein–protein interactions and associated signaling pathways intensely regulated by the intracellular oxidative state [1].

Antioxidant enzymes are the first line of protection against an excess of ROS. Superoxide dismutases (SODs) convert the superoxide radicals to hydrogen peroxide (H_2_O_2_), which is then further detoxified by catalase (CAT) and glutathione peroxidases (GPxs) [6]. In particular, GPxs are members of a common protein superfamily found in all eukaryotic cells. Recent evidence suggests that the majority of these enzymes uses glutathione as substrate, but has also specificity for thioredoxin [7,8].

Contrary to common belief, cysteine-based thioredoxin-specific GPxs are both more frequent and more ancient than other isoforms. This fact raises interesting evolutionary questions regarding oligomerization and the use of selenocysteine residue in the active site [4,7].

To analyze the physiological function of GPxs in ROS detoxification, we used the unicellular eukaryotic organism *Tetrahymena thermophila*. *T. thermophila* is a ubiquitous freshwater ciliate that has been extensively investigated and represents an ecotoxicological tool for water quality assessment, having an aquatic toxicology potency that correlates to that observed in fish [9,10,11]. This ciliate combines all the characteristics of unicellular organisms, such as high growth rate and affordable lab maintenance, with the presence of a complete eukaryotic molecular machinery, making *Tetrahymena* a model organism in the field of molecular and cell biology. It is worth mentioning here the discovery in *Tetrahymena* of catalytic RNA, a finding that had a tremendous impact on understanding the importance of RNA in cell physiology and evolution [12]. *T. thermophila*, similar to other ciliates, separates germline and somatic genomes into two distinct nuclei within a single cell. In addition, cells use UGA as the only stop codon as well as selenocysteine-specific codon. As a consequence, *Tetrahymena* is one of the very few organisms able to translate all 64 codons into amino acids [13,14]. The knowledge of the genome and the presence of pathways missing in other organisms makes *T. thermophila* an ideal model for functional genomic studies to address biological, biomedical, and biotechnological questions of more general importance [15]. 

In a previous work [16], we reported the molecular characterization of *T. thermophila* Cu,Zn SODs and studied gene expression in presence of an excess of copper (Cu), used as pro-oxidant. In fact, Cu is an essential trace element that plays a pivotal role as a catalytic cofactor for a variety of metalloenzymes. Moreover, Cu is a redox-active metal that can participate in electron transfer reactions, with the consequent production of oxidants that can oxidize cell components [17]. Cu catalyzes the formation of highly reactive hydroxyl radicals from H_2_O_2_ via the Haber–Weiss reaction and can cause the decomposition of lipid peroxides to peroxyl and alkoxyl radicals, driving the propagation of lipid oxidation [18]. Therefore, due to its chemical properties, Cu represents a good tool to study perturbations of ROS homeostasis and the role of the antioxidant system for protection of the eukaryotic molecular machinery [16,19]. Our previous data suggest that the antioxidant system of *T. thermophila* is highly specialized, and includes two unusual genes codifying for Cu,Zn SOD, *tt-sod1a,* and *tt-sod1b* [16]. Both genes are active and form an efficient detoxification pathway when the concentration of metals in the environment is high. These Cu,Zn SOD genes appear to have evolved rapidly and recently, whereas Mn and Fe SODs appear to have evolved at a relatively constant rate over the entire history of eukaryotes [20].

To add further information about the components of the antioxidant defense system of *T. thermophila*, we characterized some genes coding for GPxs, and analyzed their specific features using an integrative approach that combines wet and dry lab techniques. A text-mining was conducted to identify the potential relevance of these enzymes in their functional network, and a hand-curated pipeline written in Python to investigate the genomic features of their promoter region. Gene expression analyses and quantification of enzymatic activity after exposure to Cu were performed to support the in-silico predictions.

## 2. Materials and Methods

### 2.1. Sequence Alignment, Network, and Phylogenetic Reconstructions

Of the twelve genes encoding GPx in *T. thermophila*, we only studied four, as we were able to analyze mRNA expression only for isoforms 3, 7, 8 and 11, due to the high similarity of the their nucleotide sequences, which made it difficult to design primers that could specifically bind to each of them. The four selected GPx sequences are summarized in Table 1.

The 2000 nt upstream to coding sequence of each gene were collected from the somatic genome mapping (http://www.ciliate.org/gb2/gbrowse/Tetrahymena2020/), and analyzed using a custom Python pipeline as described in previous works [16,21] to identify putative transcription factors binding sites such as TATA box, antioxidant responsive element (ARE), half antioxidant responsive element (hARE), metal responsive element (MRE) and xenobiotic responsive element (XRE).

The database STRING [22] was used to predict the protein–protein interaction network associated with the experimentally confirmed sequences. Proteins that show a significative interaction with *T. thermophila* GPxs were clustered using Markov Cluster Algorithm (MCL) modeling (inflation parameter = 3). For each protein, the association with a specific pathway was inferred using the functional enrichment information available in the STRING database. In-silico results have been manually screened for annotation incongruences and confirmed using other databases and tools such as the Panther classification system (http://pantherdb.org) and Kyoto encyclopedia of genes and genomes (KEGG; https://www.genome.jp/kegg/pathway.html).

The T-Coffee multiple sequence alignment package (Comparative Bioinformatics Group, Barcelona, Spain) was used to obtain multiple alignment of 31 sequences from various species including other ciliated protozoa (Table S1) [23].

Statistical selection of best-fit models of nucleotide substitution were carried out using the jModelTest 2 [24] and 88 candidate models were used for this analysis.

The best-fit model of analyzed protein evolution was selected according to ProtTest 3 [25] and used 122 candidate models.

Phylogenetic trees were built using the Bayesian inference (BI) method applied in Bayes 3.2 [26] and the maximum likelihood (ML) method applied in PhyML 3.0 [27]. For the BI method, four independent runs were performed for 1,000,000 generations sampled every 1000 generations. For the ML method, bootstrap analyses were performed on 100,000 trees. The annotated phylogenetic trees were displayed using FigTree v1.3 software (GitHub, San Francisco, CA, USA).

Finally, we employed the mechanistic empirical model (MEC) [28] that accounts for the different amino acid replacement probabilities based on the previously identified model (by using ProtTest 3) empirical replacement matrix. The codon sequences were aligned by using the PRANK software [29], and the starting tree was the one obtained with the ML method.

### 2.2. Axenic Culture and Treatment of T. thermophila

*T. thermophila* control cells (strain SB210; TSC_SD00703, *Tetrahymena* Stock Center) [16] were maintained in exponential growth (20,000 cells/mL) and treated in a liquid suspension containing 0.4% protease-peptone, 0.2% yeast extract and 1% glucose. Treated cells were grown in the same medium supplemented with CuSO_4_ at 500 µM (the maximum concentration that does not affect the growth rate of the cells, verified by growth curves). Treated and control cells were harvested after 0.5, 1, 2, 4, 24, and 48 h of exposure, according to a previous study, where the accumulation of Cu in the cells throughout the experiment is also indicated [16]. Three independent experiments of exposure were conducted.

### 2.3. Isolation of Total RNA and Preparation of Cellular Extract for Enzymatic Activity

Harvested cells were divided into two samples. One was used for total RNA isolation with TRIzol (Invitrogen, Thermo Fisher Scientific, Waltham, MA, USA), and the other was reserved for preparation of the cellular extract.

RNA isolation was performed following the protocol provided by the manufacturer; however, the total RNA was further purified with LiCl 8 M in order to prevent the interference of glucoside contaminants with downstream applications such as RT-PCR and TA-cloning. The RNA quantification was performed using the ND-1000 spectrophotometer (Thermo Fisher Scientific); RNA integrity was assessed by capillary electrophoresis using the Agilent Bioanalyzer 2100, with the RNA 6000 Nano (Agilent, Santa Clara, CA, USA).

The cellular extract was prepared by washing the cells in 10 mM Tris-HCl buffer (pH 7.5) and homogenizing them in the extraction buffer (10 mM Tris-HCl, 1 mM ethylenediamine tetra-acetic acid (EDTA), 1 mM DTT, 0.5 M sucrose, and 0.15 M KCl A, pH 7.6) using a Polytron at 4 °C. The soluble protein fraction containing GPx proteins was later separated from the cellular debris by centrifugation (46,000× *g* for 50 min at 4 °C).

### 2.4. Experimental Validation of the Putative Automated mRNA Predictions

To verify the expression of annotated GPx sequences, specific primers were designed to amplify the selected number of GPx genes. Primer sequences are reported in Table S2.

ImProm II Reverse Transcriptase (Promega, Madison, WI, USA) kit was used to synthesize first strand cDNA that was then amplified with an oligo dT anchor primer (to select for polyadenylated transcript) and gene-specific primers. Amplifications were performed with 50 ng of cDNA, following the PCR program: 94 °C for 2 min, 35× (94 °C for 30 s, Tm for 30 s, 72 °C for 1 min), 72 °C for 10 min. All amplicons were gel-purified with the NucleoSpin Extract 2 in 1 (Macherey-Nagel, Düren, Germany) and ligated with the pGEM^®^-TEasy Vector (Promega). XL1-Blue *E. coli* cells were used to clone the vector. Plasmids from positive clones were sent to BMR Genomics (University of Padova) and sequenced using ABI 3730xl DNA Analyzer (Applied Biosystems, Foster City, CA, USA). Sequencing results were manually inspected and compared with the predicted putative sequences using pairwise alignments via Water (https://www.ebi.ac.uk/Tools/psa/emboss_water/).

Semi-quantitative RT-PCR analysis was then performed to quantify the transcription level of each GPx isoform. Expression levels were reported as the ratio between transcripts of GPx and the housekeeping gene 17S rRNA (relative quantification, RQ) in the same sample. Gel band relative intensities were quantified using a quantitative ladder and the Quantity One software (Gene RulerTM, Fermentas, Thermo Fisher Scientific).

### 2.5. Determination of Total and Selenium-Dependent GPx Activity

Enzymatic activity of GPx proteins was measured in cellular extracts. Although it was not possible to distinguish the activity of each single isoform, total and selenium activities were determined using a spectrophotometric methodology as previously described [30]. H_2_O_2_ was used as substrate for determination of Se-dependent activity and cumene hydroperoxide for sum of Se-dependent and Se-independent activities. The decrease in glutamate synthase (NADPH) concentration was recorded at 340 nm for 5 min. One unit of GPx activity equals 1 mol glutathione oxidized per min. Data were normalized on total protein concentration (Lowry procedure using bovine serum albumin as a standard).

### 2.6. Statistics

Statistical analyses and the distribution of the collected data were performed using the PRIMER statistical program and the Shapiro test, respectively. A one-way ANOVA, followed by the Student–Newman–Keuls test was performed to assess significant differences (*p* < 0.05).

## 3. Results

### 3.1. Gene Characterization, Evolution, and Protein–Protein Interaction Network

**Figure 1:**
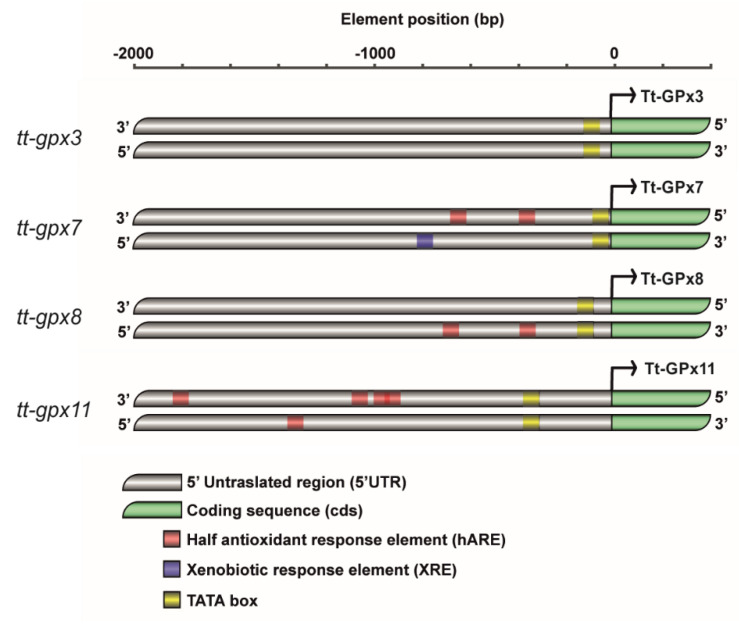
Arrangement of putative half antioxidant responsive element (hARE) and xenobiotic responsive element (XRE) in upstream regions of *tt-gpx11*, *tt-gpx3*, *tt-gpx8*, and *tt-gpx7* genes. hAREs carrying consensus sequences are indicated by red blocks, XRE and TATA-box consensus sequences are annotated as filled blue and black blocks, respectively. Transcription start site is indicated by an arrow.

The distribution of putative regulatory sequences in the four GPx genes is shown in Figure 1. In these genes, no ARE and MRE sequences were identified, while hARE sequences are present in the promoter region of *tt-gpx7*, *tt-gpx8* and *tt-gpx11*. In addition, the gene *tt-gpx7* contains an XRE motif, while no putative regulatory sequence was detected in the gene *tt-gpx3*.

**Figure 2:**
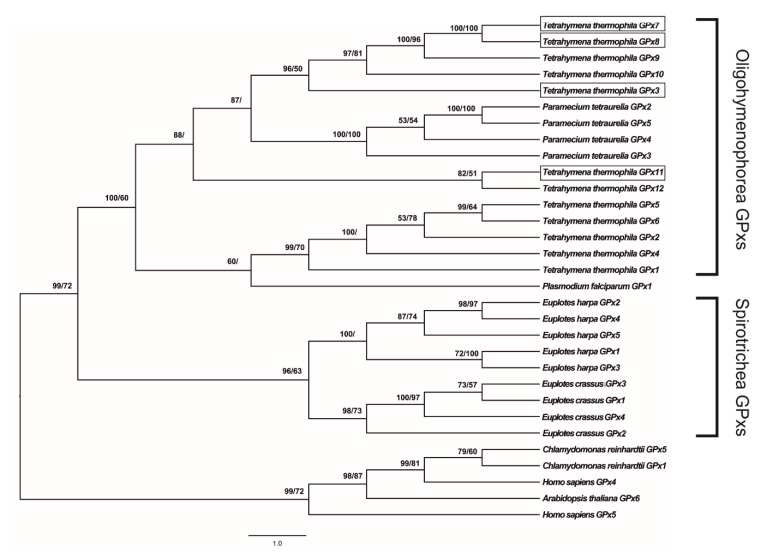
Phylogenetic relationships among glutathione peroxidases (GPxs) of various organisms reconstructed on the basis of the cDNA coding region sequences and using both Bayesian interference (BI) and maximum likelihood (ML) methods. Bayesian posterior probability (first number) and bootstrap values higher than 50% are indicated on each node, respectively. The scale for branch length (1.0 substitution/site) is shown below the tree. *T. thermophila* GPx3, GPx7, GPx8 and GPx11 are boxed.

jModelTest 2.0 software indicated that the GTR + I + G model is the best-fit model to analyze the evolution of *gpx* coding sequences, with a gamma shape value (four rate categories) of 1.688 using all statistical criteria: Akaike Information Criterion (AIC), Corrected Akaike Information Criterion (cAIC) and Bayesian Information Criterion (BIC) (−lnL = 11,911.32). Figure 2 shows the GPx phylogenetic tree generated by the application of the BI and ML methods to the data set of coding sequences.

As expected, *T. thermophila* GPxs clustered with the GPxs belonging to the class Oligohymenophorea and are separated from orthologs of other organisms, including ciliated protozoa belonging to the class Spirotrichea (posterior probability 100%, bootstrap value 60%). Within this cluster, the sequences of *T. thermophila* are distributed in two groups, one of which includes all four GPxs investigated in this study: GPx3, GPx7 and GPx8 were found to be phylogenetically related to GPx9 and GPx10 (posterior probability 96%, bootstrap value 50%); GPx11 phylogenetically related to GPx12 (posterior probability 82%, bootstrap value 51%). The same cluster also includes the sequences of *Paramecium tetraurelia*, which is clearly separated from *T. thermophila* GPxs (posterior probability 100%, bootstrap value 100%).

ProtTest3 statistical results determined the LG + G + F model as the best model to apply for the phylogenetic analysis of GPx amino acid sequences, with a gamma shape value (four rate categories) of 1.031 using all statistical criteria (−lnL = 6622.05). Figure S1 shows a GPx phylogenetic tree generated by the application of the BI and ML methods to the data set of amino acid sequences. The cladogram topology is quite similar to the previous one, but shows some important differences concerning the GPxs of *T. thermophila*. In particular, GPx3 is not closely related to GPx7 and GPx8 (posterior probability 56%). Furthermore, GPx11 is not grouped with the other GPxs of *T. thermophila*, emerging together with the GPx1 of *Plasmodium falciparum* (posterior probability 79%).

MEC analysis indicated an absence of positive selection in the evolution of the GPx sequences of the analyzed organisms (Figure S2).

**Figure 3:**
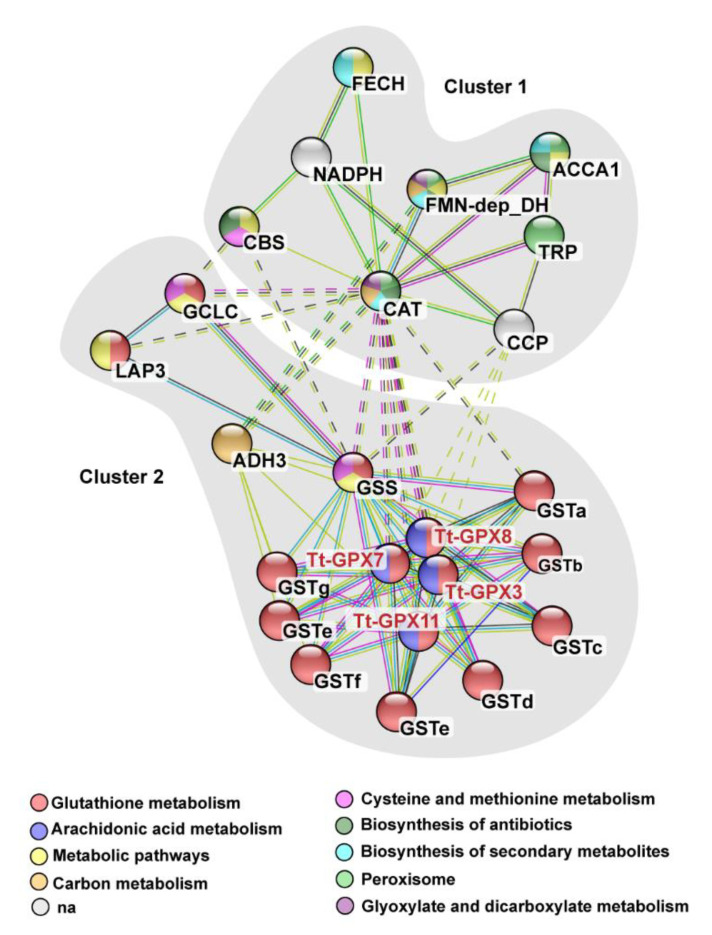
Protein–protein interaction network. The nodes in the network are color-coded in accordance with their Kyoto encyclopedia of genes and genomes (KEGG) pathway categories. The colors assigned for each KEGG pathway are indicated at the bottom of the figure. The color of the lines connecting the nodes (proteins) indicates the typology of protein–protein interaction. The interactions are classified in three groups: know (turquoise = from curated database, pink = experimentally determined interactions), predicted (green = gene neighborhood, blue = gene co-occurrence) and others (yellow = text-mining, black = co-expression). The network has been clustered using the MCL method, and the identified clusters are shown in gray. Interactions that do not belong to the same network are indicated by dashed lines. ACCA1, acetyl-coenzyme A carboxylase carboxyl transferase subunit alpha; ADH3, class III alcohol dehydrogenase; CAT, catalase; CBS, cystathionine-β-synthase; CCP, cytochrome C peroxidase; FECH, ferrochelatase; FMN-dep_DH, FMN-dependent dehydrogenase; GCLC, glutamate-cysteine ligase, catalytic subunit; GSS, glutathione synthase; GST, glutathione S-transferase; LAP3, cytosol aminopeptidase; NADPH, glutamate synthase; TRP, tetratricopeptide repeat protein.

We used text mining and PubMed literature archives to construct a network of proteins associated with Tt-GPxs (Figure 3). The proteins that show associations with Tt-GPxs are grouped in two clusters. Cluster one contains cytosolic proteins that are often associated with the oxidative metabolism. Instead, cluster two contains multiple isoforms of enzymes that have physiological relevance, since they are involved in the metabolic pathway of glutathione biosynthesis and transport (KEGG pathway n. tet00480).

### 3.2. GPx mRNA Levels

**Figure 4:**
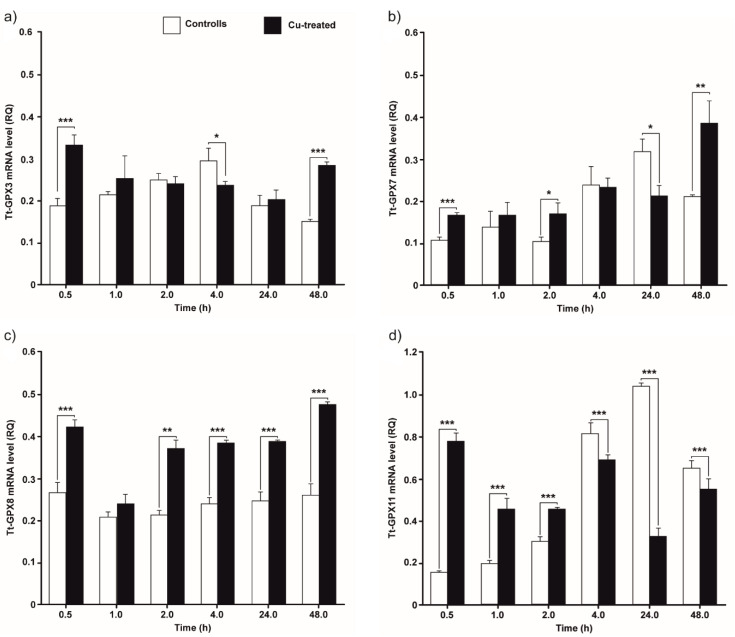
Expression levels of Tt-gpx3 (**a**), Tt-gpx7 (**b**), Tt-gpx8 (**c**) and Tt-gpx11 (**d**) GPx mRNAs in control and copper- (Cu)-treatment conditions. *T. thermophila* cells harvested at 0.5, 1, 2, 4, 24, and 48 h. The results are reported as mean of three independent experiments ± SD. Statistical differences between the two experimental groups are reported as follows: (*) *p* < 0.05, (**) *p* < 0.005 and (***) *p* < 0.001.

The accumulation of the four Tt-GPx transcripts in control (not treated) and Cu-treated cell cultures collected at different times after inoculation is shown in Figure 4.

The *tt-gpx3* mRNA level in Cu-treated cells is higher than in control cells only at 30 min and 48 h (Figure 4a; *p* < 0.001). At the other time points, no statistically significant differences in mRNA levels between treated and control cells were measured, with the exception of after 4 h of incubation, when there is a higher *tt-gpx3* expression in the control cells (*p* < 0.05).

The gene *tt-gpx7* is overexpressed after 30 min, 2 h, and 48 h of exposure to Cu while it is underexpressed after 24 h (Figure 4b; *p* < 0.05). At 1 h and 4 h of treatment, there are no statistically significant differences between treatments and controls.

The *tt-gpx8* expression is statistically higher in Cu-treated cells for almost the entire duration of the experiment (Figure 4c). Only after 1 h of treatment, there is no significant difference between treatment and control.

The mRNA expression of *tt-gpx11* in treated cells is significantly higher than controls for the first 2 h (Figure 4d; *p* < 0.001). Afterwards, this trend is reversed, with mRNA levels being statistically higher in controls between 4 h and 48 h (*p* < 0.001).

### 3.3. Total and Selenium-Dependent GPx Activity

**Figure 5:**
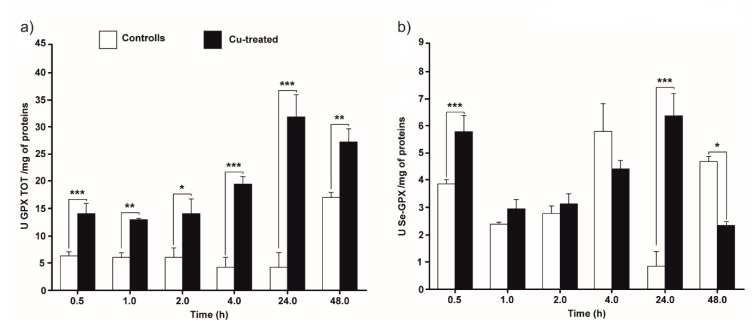
Total GPx (**a**) and selenium-dependent GPx (**b**) activities in control and Cu-treatment conditions. *T. thermophila* cells harvested at 0.5, 1, 2, 4, 24 and 48 h. The results are reported as mean of three independent experiments ± SD. Statistical differences between the two experimental groups are reported as follows: (*) *p* < 0.05, (**) *p* < 0.005 and (***) *p* < 0.001.

Figure 5 shows the time-course of the GPx activity in control and Cu-treated cultures, collected at different times of incubation. The figure shows the activity of both Se-dependent and total GPxs.

Total GPx activity in Cu-treated cultures was found to be statistically greater than in control cells throughout the experiment (Figure 5a). The graph shows a more constant trend in the first 4 h, with a statistically significant increase starting from 24 h, with values that triplicate compared to previous times (*p* < 0.05).

Se-GPx activity in the presence of Cu did not significantly differ from that of the control cultures (Figure 5b), except at 30 min and 24 h, where the activity is higher in treated cells (*p* < 0.001), and at 48 h when the activity is higher in the control cells (*p* < 0.05).

## 4. Discussion

In this paper, we characterized four of the twelve glutathione peroxidase genes annotated as predicted in the *Tetrahymena* Genome Database. Three of these genes (*tt-gpx3*, *tt-gpx7*, and *tt-gpx8*) do not contain TGA codon in the open reading frame (Figures S3–S6) and codify for non-selenium-dependent GPx (NS-GPx). In these three proteins, the four amino acids composing the catalytic core are conserved, confirming that the evolutionary process that affects the differentiation of GPx isoforms tends to preserve the amino acid composition required for the enzymatic activity.

Surprisingly, Tt-GPx11 encoded by the fourth of these genes lacks these amino acids but maintains the motif FPCNQF that is conserved in eukaryotic GPxs and has regulatory and functional proprieties. The presence of this motif can thus explain the current classification of this gene as a member of the GPx superfamily [8]. In addition, in-silico characterization of the *tt-gpx11* promoter region revealed the presence of four hAREs, which are a short version of ARE responsible for the regulation of transcription in the presence of ROS [31]. These putative regulatory elements are commonly found in the promoter regions of genes belonging to the antioxidant system and contribute to the overall regulation of transcription in the presence of ROS inducers [16,20]. These peculiar structural features, occurring only in Tt-GPx11 isoform, have suggested that this gene could originate from an ancestor with the complete catalytic tetrad and may be considered an apomorphic trait [32]. Based on the phylogenetic analysis results, Tt-gpx11 evolution does not follow the pathway of other *T. thermophila* NS-GPxs and presents some common features with the only Se-GPx (Tt-gpx12).

The phylogeny shown in Figure 2 also supports the general idea that the *T. thermophila* GPx ancestor was a NS-GPx, and the inclusion of selenocysteine in GPx is an event that occurred later during evolution [7]. Many ciliates including *Tetrahymena* are known to translate the traditional stop codons UAG and UAA as glutamine, leaving only UGA as a usable stop codon. The fact that UGA is also used to encode selenocysteine probably exerted a strong selective pressure, which severely limits the appearance of Se-GPxs in most ciliates, despite the lower catalytic efficiency of NS-GPXs [33]. Less likely, Tt-GPx11 might be the result of convergent adaptation being derived from a different ancestral protein that acquired molecular and functional characteristics typical of GPx proteins later in the evolution.

Regarding the evolution of the other GPxs in *T. thermophila*, the phylogenetic analysis supports the hypothesis that multiple gene duplications occurred over evolutionary time. The resulting increase in GPx complexity and specialization may thus contribute to the overall tolerance of this species to exogenous ROS inducers, as previously observed for metallothionein and superoxide dismutase genes [16,34]. Another interesting point is that NS-GPxs emerge grouped in two very distinct clades in the phylogenetic tree, and only the one that includes isoforms 3, 7, 8, 9 and 10 is closely related with all GPx isoforms of *P. tetraurelia*. This suggests that the gene duplications that characterizes the evolution of this protein family took place at least in two distinct moments, one before and one after the speciation event leading to differentiation of the two genera within the Spirotrichea class.

The topology of cladograms obtained by analysing amino acid and cDNA sequences is similar, though not identical. It seems that few nucleotide substitutions in the open reading frame of the *tt*-*gpx* genes are not synonymous, thus producing a little variability on the protein primary sequences. This result suggests that negative selection may have had a significant impact on the evolution of GPxs in ciliated protozoa, while ensuring the maintenance of the function of these enzymes. This evolutionary scenario is supported by results of MEC analyses. However, it is well known that positive selection is one of the primary sources of evolutionary innovation driving adaptation of species in new environments [35]. In fact, many protein families, including those involved in important cell functions, have undergone significant positive selection during their evolution [36,37]. Given that some isoforms, such as Tt-gpx11 and Tt-gpx12, represent members of the GPx family in which molecular evolution changed the ancestral structural features, it is possible to hypothesize that positive selection acted only on some portion of the protein, as suggested for some proteins of the human interactome [38]. Appropriate analyses on a larger sequence dataset are required to confirm this hypothesis.

From the functional point of view, among the proteins that show associations with Tt-GPxs, CAT and peptide-containing tratricopeptide repeat (TRP), responsible for the production and clearance of superoxide [6,39], are in the first cluster. TRP-peptides are often involved in the production of superoxide as a result of an immune response to microbial infection. Their interaction with GPx proteins might play a fundamental role in the regulation of free radical production as a mechanism of survival in animal species after immune insults, as supported by previous findings showing the cardinal role of the antioxidant system in the mechanism of immunity, especially in invertebrate models [21]. In the second cluster, proteins belonging to the glutathione synthesis pathway are grouped. This KEGG category includes proteins such as glutathione S-transferases (GST; EC 2.5.1.18) and glutathione synthetases (GSS; EC 6.3.2.3). Cu actively affects the expression of GSS and GPXs in cordate invertebrates and, therefore, these proteins are essential in the regulation of ROS homeostasis by secondary or direct consumption of de-novo synthesized glutathione. This cluster also includes the enzyme glutathione-dependent formaldehyde dehydrogenase (ADH3, EC 1.2.1.1), a cytoplasmatic enzyme that is key in the pathological increase in ROS in LPO-mediated retinopathies [40].

What does not emerge from protein–protein interaction network analysis is the correlation between GPxs and peroxiredoxins (Prdxs). Although the biological role of Prdxs is comparable to that of GPxs as scavenger against peroxides [41,42], only recently these proteins have been described in *T. thermophila* [43]. Therefore, more information is needed to better recognize the physiological role of these proteins.

Interestingly, Cu has been shown to interfere severely with ion-transport in the brain in mouse models and humans. Therefore, a wide range of neuropathies, such as Huntington, Alzheimer’s, Parkinson, and prion diseases are dependent on Cu-induced oxidative stress resulting from ion imbalance [44,45,46,47]. Other diseases that showed a pivotal role of ROS in their progression are the neurodegenerative diseases, such as frontotemporal dementia (FTD) and amyotrophic lateral sclerosis (ALS), as well as paraneoplastic syndromes [2,3,4]. This reinforces the need to gain a better understanding of GPx’s role in the eukaryotic cell physiology, since it can be a critical factor in the characterization of the complex spectrum of physiological and pathological conditions.

Gene expression analysis demonstrates, for the first time, that *T. thermophila* is able to biosynthesize a GPx with selenium-dependent activity. This is an important result because recent studies have only highlighted that mRNA for a Se-GPx is accumulated in the cell [32], and this observation does not certify the effective mRNA translation and protein synthesis. The demonstration obtained in the present work is of further interest if we consider that the mRNA sequence encoding Tt-GPx12 (the only Se-GPx of *T. thermophila*) has no detectable selenocysteine insertion sequence (SECIS) element, which represent a sequence segment in the 3’UTR region considered essential for the insertion of a selenocysteine in the primary sequence of the protein.

Another interesting observation derived from gene expression analysis is that total GPx activity increases at the end of the experiment, even in control cells, when the cellular culture is characterized by a stable growth, and the aging-derived ROS damage starts to accumulate in older cells. This phenomenon is in line with the “redox theory of aging”, a widely accepted scientific definition of a process characteristic of all biological systems, from unicellular to multicellular organisms. In fact, the decay of redox stress response capacity and the subsequent increase in cellular redox damage are elementary characteristics of aging and effect all the antioxidant systems [48,49,50]. The mRNA levels for the four considered isoforms in the absence of Cu show two different dynamics: *tt-gpx7* and *tt-gpx11* transcriptions statistically increase with time when Tt-gpx3 and Tt-gpx8 mRNA accumulation levels remain almost constant. This is probably due to a different regulation of gene transcription in response to cell growth, linked to the different roles played by the specific isoforms, a mechanism that depends on the phosphorylation cascades of cell-signaling [51,52]. The high rate of cell division could influence energy consumption and increase mitochondrial activity, the rising of H_2_O_2_ production by mitochondria and biosynthesis of antagonistic antioxidant enzymes such as Tt-gpx7 and Tt-gpx11 [52]. This finding may indicate that these two GPx isoforms are part of the first line of defense against the risk of oxidative stress in this specific physiological condition. The presence of hARE in a very high copy number in the promoter region of *tt-gpx11* gene partially confirms this hypothesis [53], but additional studies on the other *gpx* genes and their promoter regions are required in order to elucidate the effect of aging on their expression. 

Total GPx activity is significantly higher in Cu-treated cells than in control cultures. This is probably related to the amount of Cu accumulated in the cytoplasm, as described in a previous study [16]. With the currently available data, it is not possible to discriminate between the direct and the indirect effect of the metal on gene expression, due to the absence of MRE sequences in the promoter regions of *tt-gpx* genes. However, it is possible to hypothesize that the expression of these genes is favored by the increase in peroxide produced by the monoelectronic reduction of the superoxide radical (e.g., through the SOD activity) and/or by the dielectronic reduction of molecular oxygen [54]. Based on the information currently available, it is possible to exclude that the H_2_O_2_, on which the *Tetrahymena* GPxs presumably acts, comes from the action of the SODs. In fact, previous studies carried out under the same experimental conditions have shown an increase in SOD activity only in the first temporal phases of exposure to Cu [16]. To eliminate the H_2_O_2_ produced by SOD, other enzymatic systems will certainly be involved, such as CAT and Prdxs.

Regarding the accumulation of mRNA, the obtained results indicate that, in the very early stages of exposure to Cu, all four isoforms are involved in the stress response, while subsequently only Tt-gpx8 seems to play a prominent role in defenses against excessive peroxide production. In particular, *tt-gpx3* gene expression is induced by Cu although this gene does not present any conserved putative regulatory element in its promoter region. This finding reinforces previous hypothesis that in *Tetrahymena* the regulatory elements at the promoter level differ significantly from those characterized in other eukaryotes. Site-specific mutagenesis of the promoter regions of *T. thermophila* metallothionein genes supports this hypothesis by confirming that the metal-inducible expression of these genes depends on regulatory elements different from those previously described [34].

These data, in addition to hinting at a complex regulatory system that differentially involves the various GPx isoforms, highlight a gene expression feature already found in other antioxidant enzymes. In fact, comparing the increase in active protein and mRNA, the first is relatively higher than the second, suggesting that in control cells some transcripts are not immediately translated. It is known that mRNAs of stress proteins can be stored in cytoplasmic foci, such as P bodies or stress granules (SG), where they undergo degradation or future translation, respectively [55,56]. This condition is a common trait in organisms adapted to living in stressful environments [16,57], which allows them an extremely rapid response when suddenly exposed to a new stressor. Other studies will be needed to verify if this is the case also in *Tetrahymena*.

## 5. Conclusions

In conclusion, the results presented in this work confirm that the antioxidant system of *T. thermophila*, a single cell eukaryotic model species in genetic and molecular biology, presents a high degree of complexity comparable to that found in metazoan species. New information has been uncovered regarding the molecular evolution of GPxs of this species, although the evolutionary events that led to the appearance of twelve functional isoforms in *T. thermophila* will have to be further investigated. The study of gene expression revealed physiological responses that sometimes involve all the isoforms considered in this work, sometimes a single isoform. Gene expression of GPxs in our model system seems to be regulated both at the transcriptional and post-transcriptional level.

Our findings confirm that this organism is well suited to study the physiological and toxicological impacts of ROS endogenous and exogenous sources (such as aging and metals) on eukaryotic biological systems. Thus, our findings demonstrate the potential of using unicellular protists for uncovering the relationship among relevant cellular mechanisms.

## Figures and Tables

**Table 1:** List of putative sequences confirmed by cloning and sequencing and the associated IDs across *Tetrahymena* Genome Database, NCBI (RefSeq) and UniProt (UniRef) databases.

Gene	NCBI Identifier	*Tetrahymena* Genome Database ID	Uniprot ID	Predicted Isoforms Number ^1^
*tt-gpx3*	XM_001030317.2	TTHERM_01099010	EAR82654.2	GPx3
*tt-gpx7*	XM_001014606.1	TTHERM_00046110	EAR94658.1	GPx7
*tt-gpx8*	XM_001014604.1	TTHERM_00046090	EAR94389.1	GPx8
*tt-gpx11*	XM_001020136.1	TTHERM_00661720	EAR99891.1	GPx11

^1^ Prediction based on sequence identity with mammalian GPx isoforms. EMBOSS Needle software (https://www.ebi.ac.uk/Tools/psa/emboss_needle/) has been used to verify predictive auto-annotation.

## Supplementary Materials

The following are available online at https://www.mdpi.com/2076-3921/9/10/949/s1. Figure S1: Phylogenetic relationships among GPxs of various organisms, reconstructed on the basis of amino acid sequences and using both BI and ML methods; Figure S2: Detection of selective pressure on GPx using mechanistic empirical combination (MEC); Figure S3: Coding and deduced amino acid sequences of *tt-gpx3*; Figure S4: Coding and deduced amino acid sequences of *tt-gpx7*; Figure S5: Coding and deduced amino acid sequences of *tt-gpx8*; Figure S6: Coding and deduced amino acid sequences of *tt-gpx11*; Table S1: Gene sequences of GPx (and their accession numbers) used for phylogenetic reconstruction; Table S2: Sequences of primers used for PCR amplification of *T. thermophila* GPx mRNAs.
