# Supplementary material for: Molecular Characterization, Protein–Protein Interaction Network, and Evolution of Four Glutathione Peroxidases from Tetrahymena thermophila
Source: Antioxidants (Basel). 2020 Oct 2;9(10):949. doi: 10.3390/antiox9100949 (PMC7600574; doi:10.3390/antiox9100949)
Supplement: Supplementary file 1 [file antioxidants-09-00949-s001.pdf]

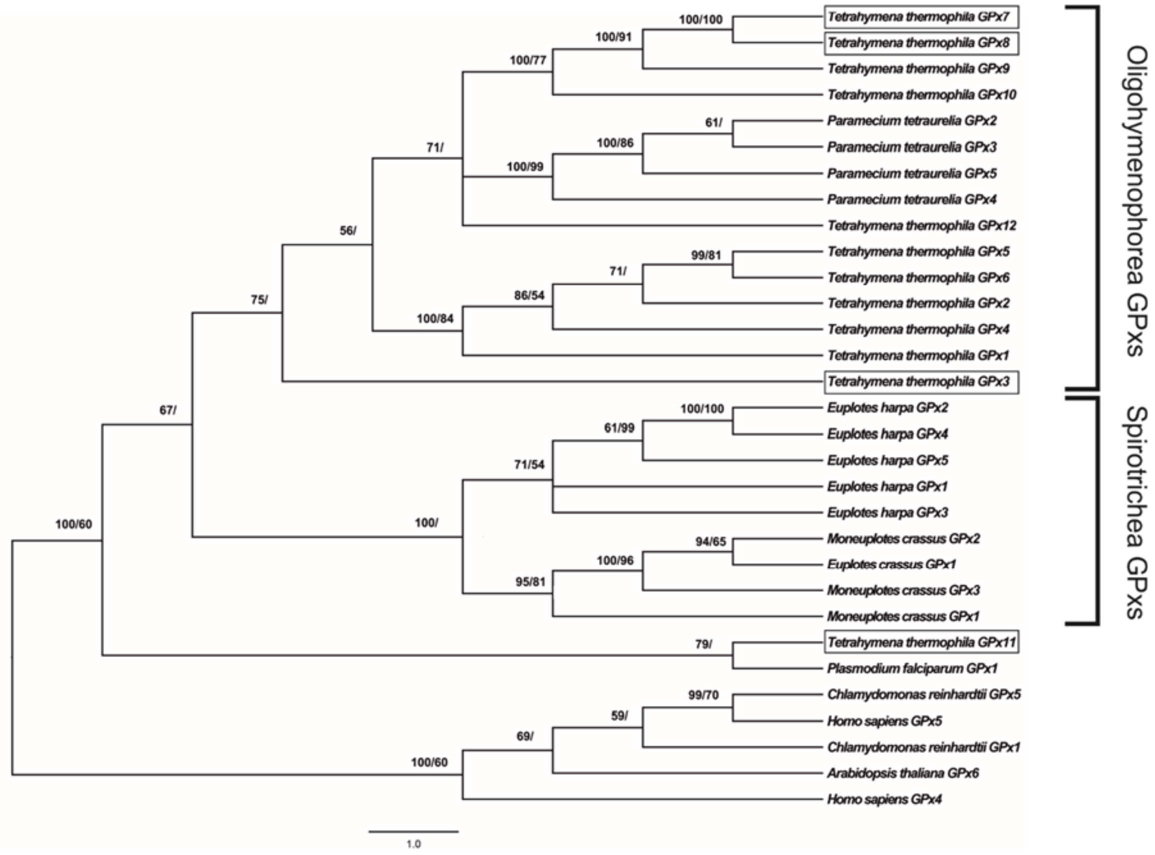

Figure S1. Phylogenetic relationships among GPxs of various organisms reconstructed on the basis of the amino acid sequences and using both Bayesian interference (BI) and maximum likelihood (ML) methods. Bayesian posterior probability (first number) and bootstrap values higher than 50% are indicated on each node, respectively. The scale for branch length (1.0 substitution/site) is shown below the tree. *T. thermophila* GPx3, GPx7, GPx8 and GPx11 are boxed.

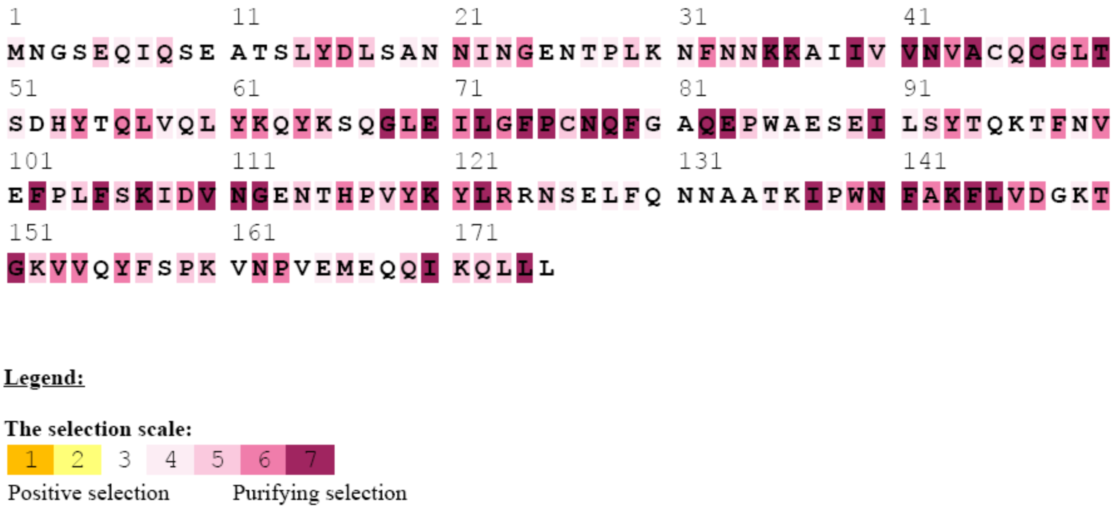

Figure S2. Detection of selective pressure on GPxs using the mechanistic empirical combination (MEC) model based mapped on *T. thermophila* GPx1. For positions with an inferred Ka/Ks > 1, a confidence interval is estimated. If the lower bound of the confidence interval is larger than 1, the inference of positive selection at this position is considered reliable and such positions are colored in dark yellow in the graphical display of the results; otherwise for position with an inferred Ka/Ks < 1, when the inference of negative selection at this position is considered reliable, such positions are colored in dark purple color.

atg gct gat aat ata aat att gca tcc ata ttt gat gtc aga gca ata gac att gat gga

M A D N I N I A S I F D V R A I D I D G

aat gag tgt caa ctt agt aaa ttt aag ggt aaa aaa gca tat tta ata gta aat gta gct

N E C Q L S K F K G K K A Y L I V N V A

agt aag tgt gga ttt act tca aca aat tat aaa cag cta tat gag ata tat aaa aac tat

S K C G F T S T N Y K Q L Y E I Y K N Y

agt gat aaa ggc tta gaa att ttg gca ttt cct tct aat taa ttt ttt aat taa gag cca

S D K G L E I L A F P S N Q F F N Q E P

ttt gat gaa cct gca att aaa gaa ttt gta aaa aaa gaa tat aat gtt gat ttt cct atg

F D E P A I K E F V K K E Y N V D F P M

ttt aaa aag att tat gtt aat gga gaa aaa aga cat gac tta tat aag tat tta gca aat

F K K I Y V N G E K R H D L Y K Y L A N

aat act cct gga ttt taa ggc tat att taa tgg aat ttc gct aaa ttt tta gtt aat gct

N T P G F Q G Y I Q W N F A K F L V N A

gag gga aaa cca gtt taa tat tat gaa cat aaa taa aat cct gta gat att gtt cct gat

E G K P V Q Y Y E H K Q N P V D I V P D

ata tta aaa tta tta aat gga gaa tga

I L K L L N G E -

Figure S3. Coding and deduced amino acid sequences of *tt-gpx3*.

atg gga tct gtt tta ctt agt tgt gtg caa aaa aaa ggc tta gaa taa att aag gtt tca

M G S V L L S C V Q K K G L E Q I K V S

cca gct aaa gat aat ttc ttt gca ttt gaa gct gtt gat aat gat ggt aaa ctt aga aag

P A K D N F F A F E A V D N D G K L R K

atg agt gag ttt aag aat aaa aaa tgc atc ctc gta gta aat gta gct tgc aag tgt gga

M S E F K N K K C I L V V N V A C K C G

tta acc tca gac cac tac aaa tag tta gtt taa att tac aaa taa tac aaa tct aga ggt

L T S D H Y K Q L V Q I Y K Q Y K S R G

ttt gaa att ctg gct ttc cca aca aat gat ttc atg gaa taa gaa cct tgg gat aat aag

F E I L A F P T N D F M E Q E P W D N K

aaa atc aaa gaa tat gtt tag act aat ttt aat gtg gat ttc tag cta ttt gat aag att

K I K E Y V Q T N F N V D F Q L F D K I

taa gtg aat gga gaa aac tgt cat gaa att tat aaa ttt tta aga ttt aac tct gaa tta

Q V N G E N C H E I Y K F L R F N S E L

cat gac tcc aag act gga aag act aga taa ata cct tgg aat ttc gct aaa ttt tta ata

H D S K T G K T R Q I P W N F A K F L I

gac cca gaa gga aaa gta gtg aaa ttc gta aac cct aaa cac aat cca gaa gtt ttg ata

D P E G K V V K F V N P K H N P E V L I

cct gat att gaa gct atg ctt aat gca tga

P D I E A M L N A -

Figure S4. Coding and deduced amino acid sequences of *tt-gpx7*.

atg gga tct gtt tta ttg agc tgc atg caa aaa agc aat tta gaa gaa gtt aag gtt tca

M G S V L L S C M Q K S N L E E V K V S

cca gct aaa gat aat ttt ttt gca ttt gaa gct att gac aat gat ggt aaa ctt aga aag

P A K D N F F A F E A I D N D G K L R K

atg agt gag ttt aag aat aaa aaa tgc atc ctc gta gta aat gta gct tgc aag tgt gga

M S E F K N K K C I L V V N V A C K C G

tta acc tca gac cac tac aaa tag tta gtt gaa att tac aaa taa tac aag tct aga ggt

L T S D H Y K Q L V E I Y K Q Y K S R G

ttt gaa att ctg gct ttc cca aca aat gat ttc atg gaa taa gaa cct tgg gat aat aac

F E I L A F P T N D F M E Q E P W D N N

aaa atc aaa gaa tat gtt tag act aat ttt aat gtt gat ttc tag cta ttt gat aag att

K I K E Y V Q T N F N V D F Q L F D K I

taa gtg aat gga gaa aat tgt cat gaa att tat aaa ttt tta aga ttt aat tct gaa ttg

Q V N G E N C H E I Y K F L R F N S E L

cat gac tcc aag act gga aag act aga taa ata cct tgg aac ttt gct aaa ttt tta ata

H D S K T G K T R Q I P W N F A K F L I

aat cca caa gga aaa gta gtg aag ttc gta agc cct aaa tac aat cca gaa gta atg ata

N P Q G K V V K F V S P K Y N P E V M I

cct gat att gaa gct atg ctt gat gct tga

P D I E A M L D A -

Figure S5. Coding and deduced amino acid sequences of *tt-gpx8*.

atg gga aat agt ggt ctg aga aag ctt ttg ttt gca gga gac atc gtc gaa tct cct tac

M G N S G L R K L L F A G D I V E S P Y

aga agc tta agc gat att aaa gtt att aat tta gat aag gaa gaa gta ttt tta gga gat

R S L S D I K V I N L D K E E V F L G D

ttg act gct aat aaa tat gca ata gta gta aat act gga tct caa aat cca aat ttt aaa

L T A N K Y A I V V N T G S Q N P N F K

tag taa atc aat gag ctc aat tag ttt aaa taa gaa aac aaa gat aaa tta gaa att ttg

Q Q I N E L N Q F K Q E N K D K L E I L

gca ttc cct tgt aat tag ttc tat aat gaa cct tca aac ttc aaa act att aaa gat tca

A F P C N Q F Y N E P S N F K T I K D S

tat tct agc ctt gta taa ttt cca gtt ttt tag aag gta gag gta aat ggt tca tac atg

Y S S L V Q F P V F Q K V E V N G S Y M

cat cct ctc tac aaa ttc ttg aag aga cat tct tct ctc tat aat tat aaa tta tta aat

H P L Y K F L K R H S S L Y N Y K L L N

gga gca aaa ata act gaa gat ttt tcc aaa ttt ctt atc aac aca aaa gga gaa gta gtt

G A K I T E D F S K F L I N T K G E V V

tca ttt tac gcc gct tca act cca ttg agt tag att taa aaa gac tta gat aca ctt aag

S F Y A A S T P L S Q I Q K D L D T L K

gcc act taa aat tga

A T Q N -

Figure S6. Coding and deduced amino acid sequences of *tt-gpx11*.

Table S1. Gene sequences of GPx (and their accession numbers) used for phylogenetic reconstruction.

SPECIES	ISOFORM	ACCESSION NUMBER
Arabidopsis thaliana	GPx6	NM_117229.4
Chlamydomonas reinhardtii	GPx1	AY051144.1
Chlamydomonas reinhardtii	GPx5	XM_001698523.1
Euplotes crassus	GPx1	MMETSP1380-20130617|3253_1
Euplotes crassus	GPx2	FJ440154.1
Euplotes crassus	GPx3	JN872737.1
Euplotes crassus	GPx4	FJ440155.1
Euplotes harpa	GPx1	MMETSP0213-20121227|20483_1
Euplotes harpa	GPx2	MMETSP0213-20121227|20569_1
Euplotes harpa	GPx3	MMETSP0213-20121227|20483_1
Euplotes harpa	GPx4	MMETSP0213-20121227|20569_1
Euplotes harpa	GPx5	MMETSP0213-20121227|13322_1
Homo sapiens	GPx4	BC011836.2
Homo sapiens	GPx5	AJ005277.1
Paramecium tetraurelia	GPx2	XM_001443233.1
Paramecium tetraurelia	GPx3	XM_001423564.1
Paramecium tetraurelia	GPx4	XM_001449625.1
Paramecium tetraurelia	GPx5	XM_001442917.1
Plasmodium falciparum	GPx1	XM_001350492.1
Tetrahymena thermophila	GPx1	XM_001022734.1
Tetrahymena thermophila	GPx2	XM_001031216.4
Tetrahymena thermophila	GPx3	XM_001030317.2
Tetrahymena thermophila	GPx4	XM_001031214.1
Tetrahymena thermophila	GPx5	XM_001031215.1
Tetrahymena thermophila	GPx6	XM_001031213.1
Tetrahymena thermophila	GPx7	XM_001014606.1
Tetrahymena thermophila	GPx8	XM_001014604.1
Tetrahymena thermophila	GPx9	XM_001011054.3
Tetrahymena thermophila	GPx10	XM_012796335.1
Tetrahymena thermophila	GPx11	XM_001020136.1
Tetrahymena thermophila	GPx12	XM_012798148.1

Table S2. Primers designed with PRIMER3 and used for RT-PCR.

ID	Primer Forward	Primer Reverse
TTHERM_ 00661720	5'-ATAGTGGTCTGAGAAAGCTTTTGTTTGCAGG-3'	5'-CATGTATGAACCATTTACCTCTACCTTCT-3'
TTHERM_ 01099010	5'-TTGATGTCAGAGCAATAGACATTGATGGA-3'	5'-CTGGTTTTCCCTCAGCATTAACT-3'
TTHERM_ 00046090	5'-GAGCTGCATGCAAAAAAGCAATTTAGAAG-3'	5'-GTAGTGGTCTGAGGTTAATCCACAC-3'
TTHERM_ 00046110	5'-TAGTTGTGTGCAAAAAAAAGGCTTAGAAT-3′	5′-'TCACTACTTTTCCTTCTGGGTCT-3'
17s	5′-AAATGTTTACTCCCTAAGTCGAACC-3′	5′-CCTGAGAAACGGCTACTACAACTAC-3′
